# Supplementary material for: Isotopic evolution of planetary crusts by hypervelocity impacts evidenced by Fe in microtektites
Source: Nat Commun. 2021 Sep 22;12:5646. doi: 10.1038/s41467-021-25819-6 (PMC8458397; doi:10.1038/s41467-021-25819-6)
Supplement: Supplementary file 1 — Supplementary Information [file 41467_2021_25819_MOESM1_ESM.pdf]

Supplementary materials for "Isotopic evolution of the planetary crusts by hypervelocity impacts evidenced by Fe in microtektites"

Authors

S. M. Chernonozhkin[1]*, C. González de Vega[1], N. Artemieva[2,3], B. Soens[4], J. Belza[1], E. Bolea-Fernandez[1], M. Van Ginneken[5], B. P. Glass[6], L. Folco[7,8], M. J. Genge[9], Ph. Claeys[4], F. Vanhaecke[1], S. Goderis[4]

[1] Atomic & Mass Spectrometry – A&MS Research Unit, Department of Chemistry, Ghent University, Campus Sterre, Krijgslaan 281 – S12, BE9000 Ghent, Belgium.

[2] Planetary Science Institute, Tucson, AZ 85719, USA.

[3] Institute for Dynamics of Geospheres RAS, 117334 Moscow, Russia.

[4] Analytical, Environmental, and Geochemistry, Vrije Universiteit Brussel, Pleinlaan 2, BE1050 Brussels, Belgium.

[5] Centre for Astrophysics and Planetary Science, School of Physical Sciences, Ingram Building, University of Kent, Canterbury CT2 7NH, United Kingdom.

[6] Department of Earth Sciences, University of Delaware, Newark, DE, 19716, USA.

[7] Dipartimento di Scienze della Terra, Università di Pisa, 56126 Pisa, Italy.

[8] CISUP, Centro per l'Integrazione della Strumentazione dell'Università di Pisa, 56126 Pisa, Italy.

[9] IARC, Department of Earth Science and Engineering, Imperial College London, Exhibition Road, London SW7 2AZ, UK.

This file contents:

Supplementary note 1. Potential reasons for isotopically light Fe in microtektites, other than condensation

Supplementary Figure 1. Isotope fractionation of Fe in microtektites versus concentration of FeO in linearized coordinate space.

Supplementary table 1. Fe isotopic composition of the Australasian tektites and microtektites studied.

Supplementary note 1. Potential reasons for isotopically light Fe in microtektites, other than condensation

Surprisingly light Fe isotopic signatures with $\delta^{56/54}$Fe values down to -2.85‰, observed for microtektite 137-8 from the V19-153 location in the Eastern Indian Ocean, cannot be explained based on a continuously evaporating melted reservoir. To account for the Fe isotopic compositions of all Australasian microtektites from a single evaporation trend, fractionation factors α from 0.986 to 0.998 and a non-natural initial composition with approximate values of $\delta^{56/54}$Fe = -3‰ and FeO = 9 wt% are required. Such scenario leaves isotopically heavy and high-FeO areas of the diagram underrepresented. The light signatures found in some microtektites are also unlikely to represent initial isotopic heterogeneity in the target material,[48] because most terrestrial (igneous) rocks are characterized by $\delta^{56/54}$Fe values close to zero.[18] Although some sulfides[49] and carbonates[50,51] may be characterized by light Fe isotopic values, a $\delta^{56/54}$Fe of -2.85‰ constitutes an exceptionally low value, which would require unrealistically large contributions of such mineral phases to the target based on mass balance considerations. Although the crater associated to the Australasian field is yet to be found, a sulfur- or carbonate-rich target has not been suggested based on the geochemistry and Fe isotopic signatures of macroscopic tektites. The light Fe isotopic composition in Australasian microtektites can also not result from equilibration with sea water ($\delta^{56/54}$Fe = 0 to +1‰[82]), because lower $\delta^{56/54}$Fe values would be expected for Fe-poor particles in the case of a diffusive kinetic control. The major and trace element concentrations measured in this work and reported in literature[12,16,17,45] reflect the heterogeneous composition of a stratified target and suggest that limited to no alteration affected the microtektites studied in this work. Antarctic microtektites are in general highly pristine and only in a few exceptional cases, pits filled with palagonite are observed at the surface of the spherules, which cannot affect the Fe isotope ratios measured with LA-MC-ICP-MS in the inner parts of the particles. Australasian microtektites also show no evidence of hydration rinds, as observed for obsidian, which would be the first alteration step. The concentrations of the LREE, Sr and Ba, elements that are mobilized easily,[83] reveal linear trends that suggest only limited alteration during the residence of these particles in pelagic or other surface environments (Fig. 2). The observed trends based on REE patterns, Eu anomalies and Zr/Hf ratios relate all Australasian microtektites and macroscopic tektites to a single non-homogeneous target with a complex stratigraphy, with variable Rb/Sr ratios (Fig. 2).

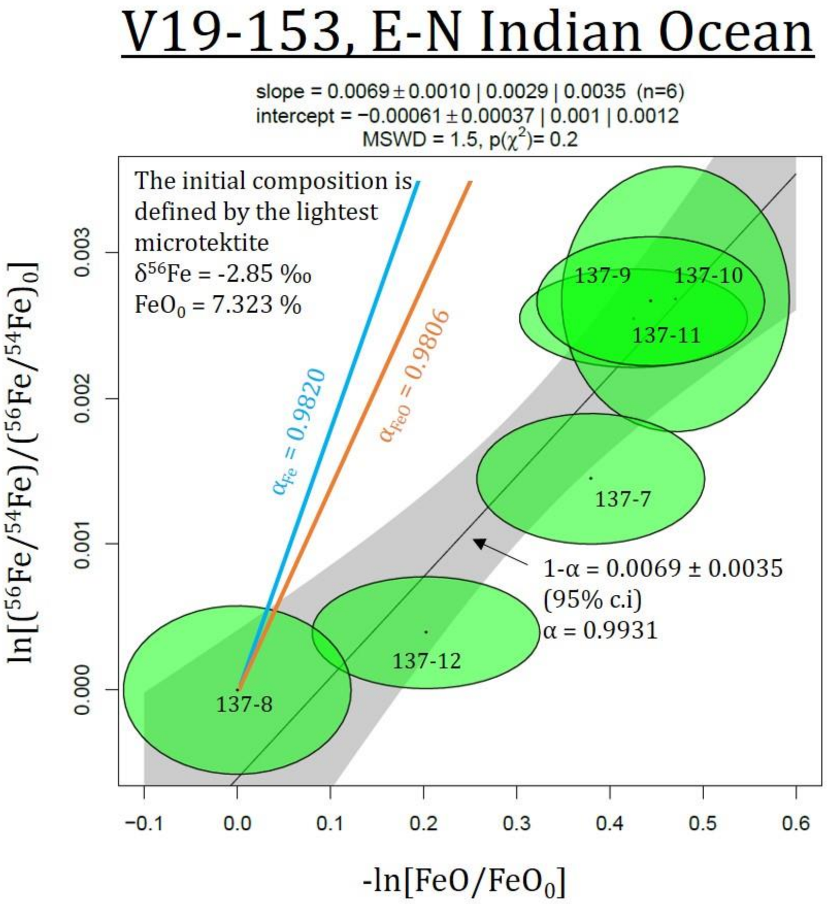

Supplementary Figure 1. Linearization of the Rayleigh distillation plot. Isotope fractionation of Fe in microtektites *versus* concentration of FeO, V19-153 collection site, East-North Indian Ocean, is plotted in such a coordinate space that Rayleigh distillation plots as a straight line with its slope equal to 1-α. The initial $\delta^{56/54}Fe_0$ and FeO_0 are defined by the lightest microtektite 137-8. The Rayleigh fractionation trends under ideal conditions of evaporation to vacuum predicted by the Hertz–Knudsen equation for Fe and FeO are shown with blue and orange lines, respectively. The regression line for Fe fractionation in the V19-153 microtektites has a slope corresponding to α = 0.9931±0.0035, significantly different from the ideal distillation to vacuum. For the microtektites from the other collection sites either the spread in the FeO concentration is not sufficiently large, or the data scatter is too high to provide a statistically meaningful linear correlation. The regression line is calculated using Model 2 of Isoplot online with overdispersion, the uncertainty ellipses represent 2SD.

Sample name	$\delta^{56/54}Fe$	*1SD*	$\delta^{57/54}Fe$	*1SD*
SRM#1	0.77	*0.07*	1.21	*0.16*
SRM#2	2.00	*0.06*	3.07	*0.16*
SRM#3	1.04	*0.09*	1.52	*0.10*
SRM#4	1.91	*0.05*	2.99	*0.18*
SRM#5	2.19	*0.09*	3.15	*0.21*
SRM#6	1.02	*0.11*	1.58	*0.19*
SRM#7	1.72	*0.06*	2.68	*0.09*
4.6	-0.24	*0.08*	-0.33	*0.13*
20.3	1.05	*0.08*	1.68	*0.13*
20.14	1.13	*0.08*	1.74	*0.13*
23.01	0.59	*0.05*	*0.82*	*0.10*
20.17	1.24	*0.07*	*1.92*	*0.11*
7.04	0.75	*0.06*	*1.14*	*0.09*
#1-1	*1.53*	*0.19*	*2.21*	*0.29*
#2-1	*0.91*	*0.09*	*1.21*	*0.21*
#2-2	*0.73*	*0.08*	*1.17*	*0.19*
#3-1	*0.32*	*0.07*	*0.43*	*0.27*
#3-2	*-0.30*	*0.21*	*-0.42*	*0.36*
ODP#1	-0.46	*0.05*	-0.64	*0.09*
ODP#3	-0.29	*0.07*	-0.41	*0.04*
ODP#4	-0.23	*0.05*	-0.31	*0.03*
17	0.59	*0.08*	0.95	*0.13*
15	-0.27	*0.08*	-0.37	*0.13*
38	0.08	*0.03*	0.13	*0.05*
37	0.02	*0.06*	0.02	*0.08*
36	0.29	*0.07*	0.49	*0.10*
35	-0.04	*0.02*	-0.05	*0.07*
137-12	*-2.45*	*0.08*	*-3.54*	0.12
137-11	*-0.31*	*0.07*	*-0.43*	*0.11*
137-10	*-0.17*	*0.19*	*-0.28*	*0.18*
137-9	*-0.19*	*0.09*	*-0.33*	*0.12*
137-8	*-2.85*	*0.12*	*-4.14*	*0.17*
137-7	*-1.40*	*0.09*	*-1.94*	*0.14*
137-6	*-0.12*	*0.29*	*-0.23*	*0.18*
137-5	*-0.93*	*0.24*	*-1.63*	*0.28*
137-4	*-0.49*	*0.33*	*-0.64*	*0.24*
137-2	*-0.95*	*0.07*	*-1.39*	*0.11*
137-1	*0.08*	*0.09*	*0.12*	*0.10*
197-1	*-0.50*	*0.09*	*-0.64*	*0.17*
197-2	*-0.21*	*0.11*	*-0.47*	*0.19*
197-3	*-0.57*	*0.08*	*-0.87*	*0.14*
197-4	*-0.06*	*0.29*	*0.13*	*0.41*
197-5	*0.60*	*0.16*	*1.10*	*0.41*
197-6	*-1.11*	*0.17*	*-1.42*	*0.32*
463-3	*-2.61*	*0.08*	*-3.77*	*0.14*
463-4	*-1.38*	*0.11*	*-1.98*	*0.20*
463-5	*-1.86*	*0.08*	*-2.75*	*0.18*
188-3	*-0.68*	*0.08*	*-1.15*	*0.14*
Macroscopic tektites				
PMJ-20	-0.09	*0.08*	-0.15	0.09
FT-598	0.00	*0.06*	0.03	*0.05*
FT-118	0.02	*0.04*	0.04	*0.06*
TI8A	-0.02	*0.03*	-0.04	*0.04*
TI8B	-0.02	*0.03*	-0.02	*0.04*
JAV-32	-0.12	*0.02*	-0.16	*0.04*
MA-142	-0.30	*0.09*	-0.43	*0.10*
MA-142	-0.03	*0.05*	-0.05	*0.07*
Philippinite VUB	-0.07	*0.03*	-0.10	*0.10*
JAV1.1	-0.28	*0.03*	-0.41	*0.06*
JAV1.5	-0.18	*0.05*	-0.27	*0.10*
JAV1.8	0.14	*0.04*	0.26	*0.17*
JAV0.9	-0.19	*0.04*	-0.25	*0.09*
Saigon BT-2	-0.22	*0.02*	-0.39	*0.12*

Supplementary table 1. Fe isotopic composition of the Australasian tektites and microtektites studied in this work.
