# Supplementary material for: Isotopic evolution of planetary crusts by hypervelocity impacts evidenced by Fe in microtektites
Source: Nat Commun. 2021 Sep 22;12:5646. doi: 10.1038/s41467-021-25819-6 (PMC8458397; doi:10.1038/s41467-021-25819-6)
Supplement: Supplementary file 3 — Description of Additional Supplementary Files [file 41467_2021_25819_MOESM3_ESM.pdf]

Description of Additional Supplementary Files

File name: Supplementary Data 1
Description: Numeric data of Fe isotopic composition and trace elemental composition of the Australasian tektites and microtektites.
